# Gastroesophageal reflux disease and non-alcoholic fatty liver disease: a two-sample Mendelian randomization combined with meta-analysis

**DOI:** 10.1038/s41598-024-63646-z

**Published:** 2024-06-02

**Authors:** Xuan Leng, Wan-Zhe Liao, Fen-Ping Zheng

**Affiliations:** 1 Department of Endocrinology, Zhejiang University School of Medicine Sir Run Run Shaw Hospital, Hangzhou, 310000 Zhejiang Province China; 2 Zhejiang University School of Medicine, Hangzhou, 310006 China; 3 Department of Clinical Medicine, The Nanshan College of Guangzhou Medical University, Guangzhou, 511436 China

**Keywords:** Gastroesophageal reflux disease (GERD), Non-alcoholic fatty liver disease (NAFLD), Mendelian randomization (MR), Meta-analysis, Causal association, Genetics, Diseases, Endocrinology, Gastroenterology, Risk factors

## Abstract

Accumulating evidence from observational studies have suggested an association between gastroesophageal reflux disease (GERD) and non-alcoholic fatty liver disease (NAFLD). However, due to that such studies are prone to biases, we imported Mendelian randomization (MR) to explore whether the causal association between two diseases exsit. Hence, we aimed to analysis the potential association with MR. The single nucleotide polymorphisms (SNPs) of GERD were retrieved from the genome-wide association study dataset as the exposure. The SNPs of NAFLD were taken from the FinnGen dataset as the outcome. The relationship was analyzed with the assistance of inverse variance weighted, MR-Egger, and weighted median. We also uitilized the MR-Egger intercept, Cochran’s Q test, leave-one-out analysis, MR-PRESSO, and Steiger directionality test to evaluate the robustness of the causal association. The meta-analysis were also implemented to give an overall evaluation. Finally, our analysis showed a causal relationship between GERD and NAFLD with aid of MR and meta-analysis (OR 1.71 95% CI 1.40–2.09; *P* < 0.0001).

## Introduction

Gastroesophageal reflux disease (GERD) is regarded as one of the most common gastrointestinal (GI) diseases at present, characterized by the backflow of acid into the esophagus which can cuase heartburn and regurgitation and futher impair the quality of patients’ life^1,2^. Medication with proton pump inhibitors (PPIs) is the routine therapy, which, however, can only alleviate the manifestation of GERD instead of eradicating the disease^3^. GERD is a common disease around the world, and the evidence shows the incidence is still increasing, especially in America and Asia, with the incidence being up to about 23% in North America and 11.7% in Asia^4,5^.

Non-alcoholic fatty liver disease (NAFLD) refers to a condition distinguished by the accumulation of fat in the liver that is not related to inordinate alcohol consumption and any specific etiological liver diseases, which is the principle cause of chronic hepatic diseases and poses a huge burdern on the national health and economy. The diagnosis of NAFLD is not clear, but liver biopsy can be extremely helpful^6^. NAFLD is often seen as the precursor of the metabolic syndrome. The most frequent risk factors of NAFLD involve obesity, hypertension, type II diabetes mellitus (T2DM), dietary habits, physical activity, and socioeconomic factors^7,8^.

A lot of cohort and observational studies have displayed that people suffering from NAFLD had a higher susceptibility to GERD, and one clinical trial indicated that the risk of reflux esophagitis would increase 16% among people with NAFLD (OR 1.16 95% CI 1.13–1.20) as compared to those without NAFLD^9–12^. In addition, a meta-analysis showed that the incidence of NAFLD was higher in people with GERD compared with healthy individuals, with an amalgamated OR of 2.07 (OR 2.07 95% CI, 1.54–2.79)^13^. However, the evidence for these associations was inconsistent, a cohort study found no clear significance between two diseases^14^. Another study suggested that GERD is the consequence, but not the cause of NAFLD^15^. Nonetheless, there is a paucity of studies investigating the causation between GERD and NAFLD.

Routine observational studies are prone to biases, such as reverse causality and residual confounding, owing to that conventionally measured exposures are often disturbed by a variety of behavioral and physiological factors^16^. Mendelian randomization (MR), a statistical approach to explore the causation between the exposure and the outcome, utilizes genetic variants as instrumental variables (IVs) to make causal inferences about the effect of an exposure on an outcome. MR takes advantage of randomization to avoid confounding errors due to the genetic variants are randomly assigned and are not influenced by confounding factors^16–18^. In addition, it is clear that the direction of causality is from the genetic polymorphism to the outcome in the MR analysis. Furthermore, a meta-analysis is a statistical method that combines the results of different studies about the same topic, which somewhat can solve conflicts. The major advantage relies on its ability of offering a precise and overall result as well as assessing potential sources of heterogeneity^19^. Therefore, we aimed to employ the MR and meta-analysis to elucidate whether there was a causal association between GERD and NAFLD, and if so, to estimate the magnitude of the causal effect.

## Materials and methods

### Data sources and selection of instrumental variables

The genome-wide association study (GWAS) dataset and the FinnGen dataset were used to perform the MR analysis. To sum up recent available data, We searched the GWAS data of gastroesophageal reflux disease (GERD) from the GWAS (https://www.ebi.ac.uk/) and FinnGen database (https://www.finngen.fi/en)^20–22^ and finally got a total of 10 groups of data for next analysis.

(Supplemental Table 1). The data of NAFLD were gained from FinnGen database which included 2275 cases and 375,002 controls, and we retrieved 20,170,233 SNPs from it. All datasets are obtained from European to avoid the potential deviation due to population stratification.

**Figure 1:**
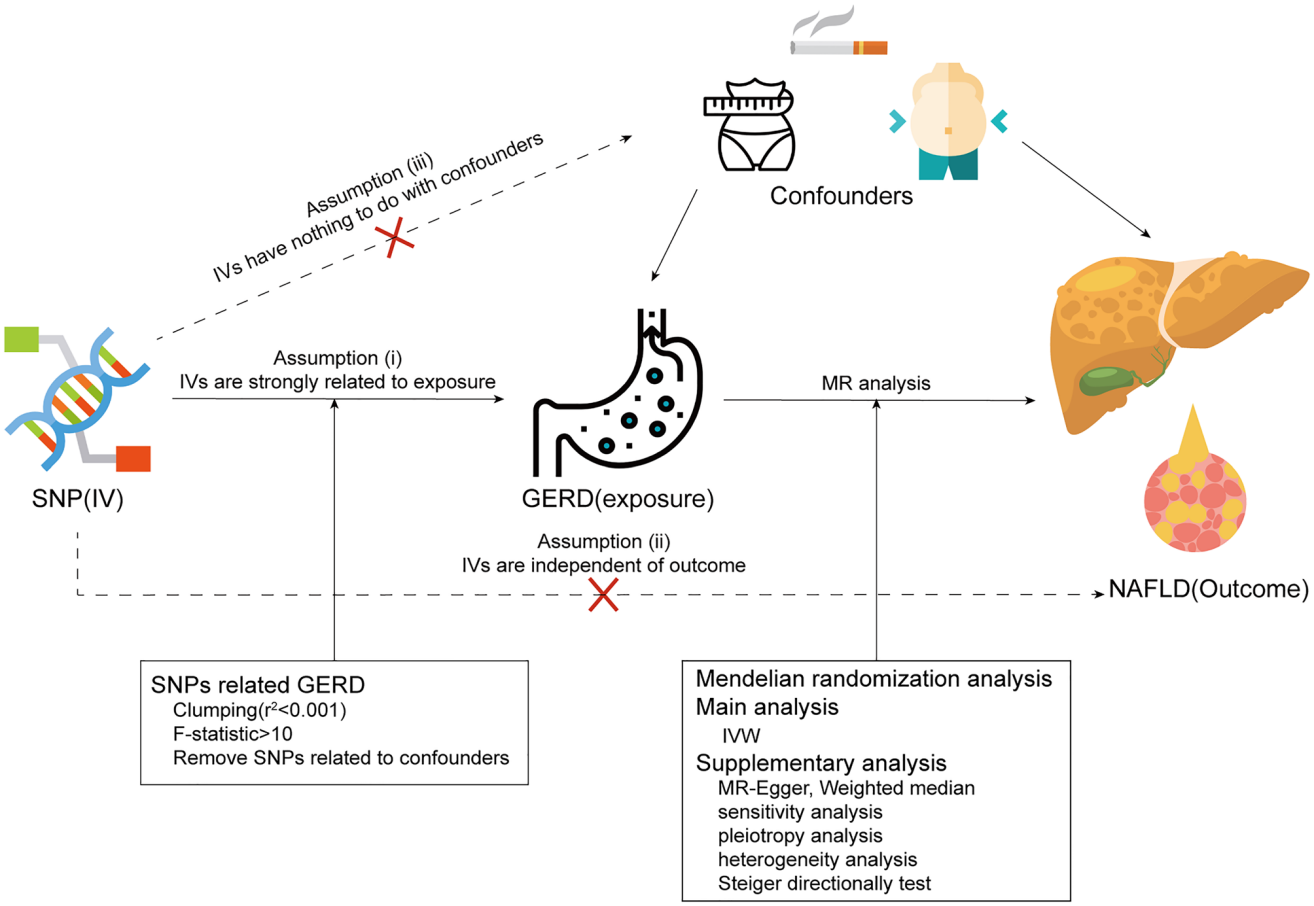
Graphical dipiction of the study design and MR assumptions in a two-sample MR design: (i) relevance; (ii) exclusiveness; (iii) independence.

Mendelian randomization analysis requires IVs to meet three significant assumptions: (i) the IVs are strongly related to exposure (relevance); (ii) the IVs are independent of outcome (independence); (iii) the IVs have nothing to do with confounders (exclusion restriction)^18,19,23^ (Fig. 1). First, we selected single nucleotide polymorphisms (SNPs) of GERD that met the standard of genome-wide significance (*P* < 5 × 10^–8^). The F-statistic is calculated and F > 10 is required to ensure the strength of the relationship between IVs and phenotype (exposure)^24^. Besides, SNPs that may have linkage disequilibrium (LD) were removed (R^2^ > 0.001) due to consideration of the violation for the independence of genetic variants^25,26^. In addition, we also eliminated genetic variants of palindromic and incompatible alleles in the process of harmonizing exposure and outcome. By the search of GWAS database (http://www.phenoscanner.medschl.cam.ac.uk), we aimed to run out on the confounder-associated SNPs according to assumption (iii) with a threshold of *P* < 1 × 10^–6^.

### Statistical analysis for Mendelian randomization

To analyze the possible causal effects between GERD and NAFLD, we chose the Inverse Variance Weighted (IVW) method as our leading strategy. In the presence of multiple genetic variants, the IVW can account for each genetic variant by averaging their effects to provide an overall causal estimate^16^. MR-Egger extends the IVW method by incorporating the analysis of horizontal pleiotropy through MR-Egger intercept^27^. The weighted median method can provide valid MR estimates even when up to half of the SNPs violate the InSIDE assumption^28^. The results of simple mode and weighted mode can be consistent even if the majority of instruments are invalid. The power of them is smaller than IVW and weighted median, but lager than MR-Egger^29^. Therefore, we can see that the other four methods (MR-Egger, Weighted median, simple mode, and weighted mode) can take effect in a looser scene, but give up some statistical power. Accodingly, we regarded them as supplements, but only when the directions of these five methods remain consistent could the MR analysis be rendered meaningful.

To test the robustness of the MR findings, heterogeneity and pleiotropy test are indispensable. We imported Cochran’s Q test to calculate the heterogeneity of the IVW. A leave-one-out analysis was also performed to test whether the potential causal association between exposure and outcome could be seriously confounded by a single SNP. MR-PRESSO and MR-Egger were implemented to test pleiotropy. MR-Egger intercept analysis was aimed to check the average pleiotropic effect, which revealed that if there was a non-zero intercept, the IVW analysis may not hold water due to pleiotropy. Only P values of above checking methods all over 0.05 rendered the conclusion effective. Finally, in order to avoid reserve causation, Steiger directionality test was utilized to ensure the unidirectionality of MR findings^30^.

All MR analysis were two-sided and performed on the platform of Rstudio (version 4.1.3) with the TwoSampleMR package (version 0.5.8). Full documentation was able to be retrieved at https://mrcieu.github.io/TwoSampleMR. The study design is shown in Fig. 1.

### Statistical analysis for meta-analysis

For the three groups derived from MR analysis, we then performed meta-analysis to evaluate summarized odds ratio (OR) and 95% confidence intervals (CI). If the heterogeneity existed, the OR would be calculated via random-effects models, otherwise fixed-effects models. We opted to use I-square tests to test heterogeneity. Only when I^2^-value < 50% and *P* > 0.05 could the result of meta-analysis be deemed as homogeneous.

The meta-analysis was performed on the platform of Rstudio (version 4.1.3) with the meta package.

### Ethical approval

No additional ethics approval was needed because all data in the study was previously collected, analyzed, and published.

## Result

### Instrumental variables for MR

After screening for genome-wide significance (*P*-value), LD (R^2^ < 0.001), and the evaluation of F (F > 10), only three groups of data of exposure was reserved for MR and meta-analysis (Table 1). The other seven sets were abandoned because the value of F does not satisfy the requirement. Moreover, to include more SNPs that contributed to GERD, a relatively relaxed threshold were applied with *P* < 5 × 10^–7^ for the Group 2 (GWAS ID: ebi-a-GCST90018848) and *P* < 5 × 10^–6^ for the Group 3 (GWAS ID: finn-b-K11_REFLUX), which had previously utilized in many MR researches^31^. We also removed SNPs that were associated with confounders of NAFLD according to literatures (obesity, hypertension, type II diabetes mellitus, dietary habits, physical activity, and socioeconomic factors)^8^. Lastly, suitable IVs were generated (Group 1: 22 SNPs; Group 2: 7 SNPs; Group 3: 29 SNPs) for the next MR analysis (Supplemental Table 2).

**Table 1:** Description of the data sources of exposure.

Group	GWAS ID	Year	Trait	Population	ncase	ncontrol	Number of SNPs
1	ebi-a-GCST90000514	2021	Gastroesophageal reflux disease	European	473,524	473,524	2,320,781
2	ebi-a-GCST90018848	2021	Gastroesophageal reflux disease	European	32,957	434,296	24,173,002
3	finn-b-K11_REFLUX	2021	Gastro-oesophageal reflux disease	European	13,141	189,695	16,380,425

### Mendelian randomization analysis

The IVW analysis displayed GERD was associated with a higher morbidity of NAFLD (Group 1: OR = 1.81; 95%CI 1.15–2.83; *P* = 0.0098; Group 2: OR = 1.94; 95%CI 1.15–3.27; *P* = 0.013; Group 3: OR = 1.63; 95%CI 1.28–2.09; *P* < 0.0001). This result was consistent with the results of weighted median (WM) (Group 1: OR = 2.35; 95%CI 1.26–4.39; *P* = 0.0075; Group 2: OR = 2.24; 95%CI 1.15–4.36; *P* = 0.018; Group 3: OR = 1.75; 95%CI 1.23–2.49; *P* = 0.0019). The MR-Egger showed no association (all *P* > 0.05). The directions all kept consistent. Cochran’s Q test of IVW and MR Egger showed no heterogeneity between IVs (all *P* > 0.05), and the results of MR-Egger intercept analysis and MRPRESSO approved no horizontal pleiotropy existed (all *P* > 0.05) (Table 2 and Supplemental Tables 3, 4, 5, 6).

**Table 2:** The heterogeneity and pleiotropy test of the MR analysis.

Expsoure (GERD)	Outcome	Methods	Heterogeneity	Pleiotropy		Directionality
				MR-Egger intercept	MR-PRESSO	
ebi-a-GCST90000514	NAFLD	MR Egger	0.37	0.88	0.44	5.24e−38
		IVW	0.43			
ebi-a-GCST90018848		MR Egger	0.85	0.97	0.92	9.12e−13
		IVW	0.92			
finn-b-K11_REFLUX		MR Egger	0.78	0.20	0.76	8.19e−70
		IVW	0.74			

**Figure 2:**
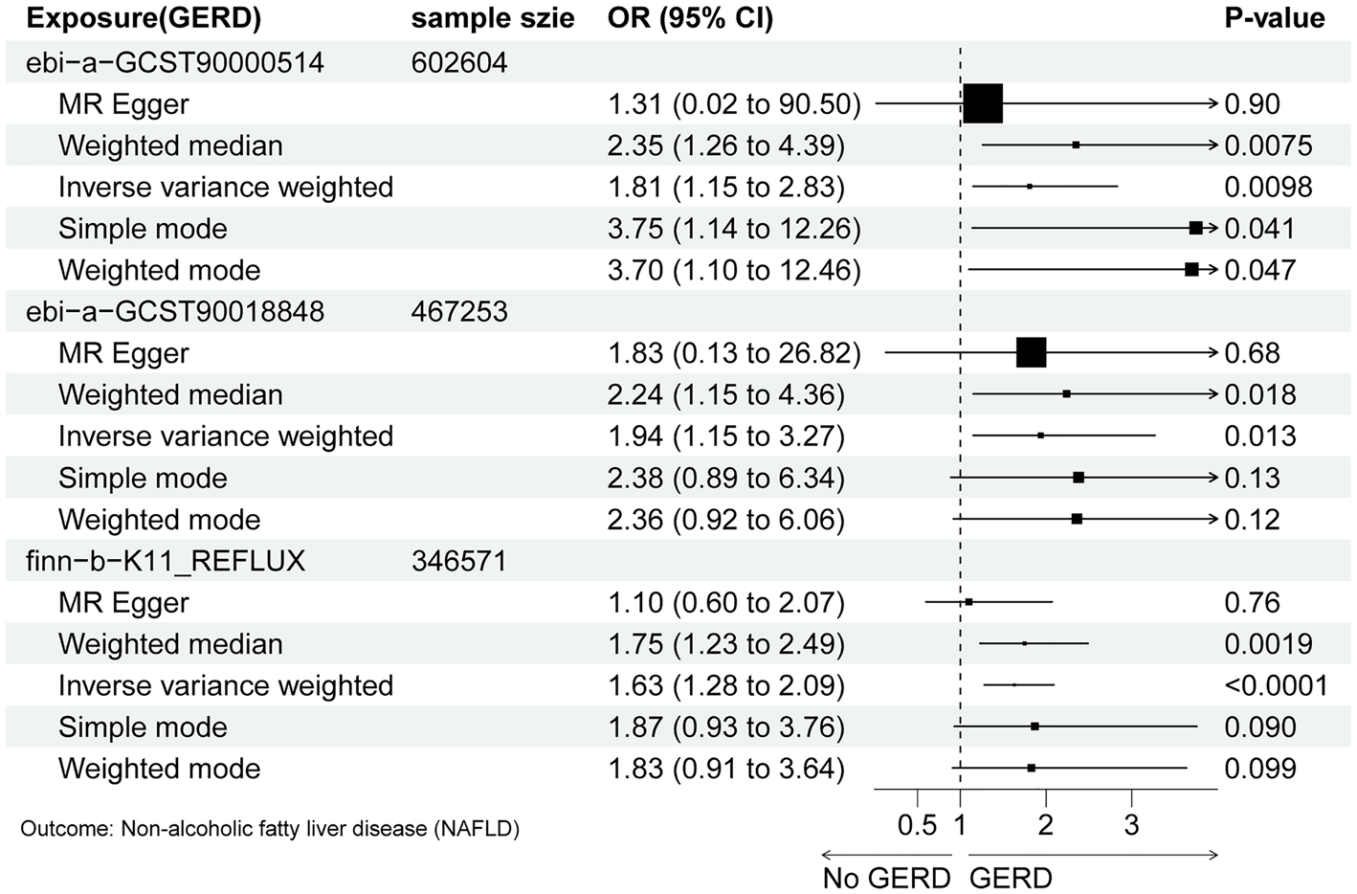
The MR results of the causal effect of GERD on NAFLD (with Forest plot).

**Figure 3:**
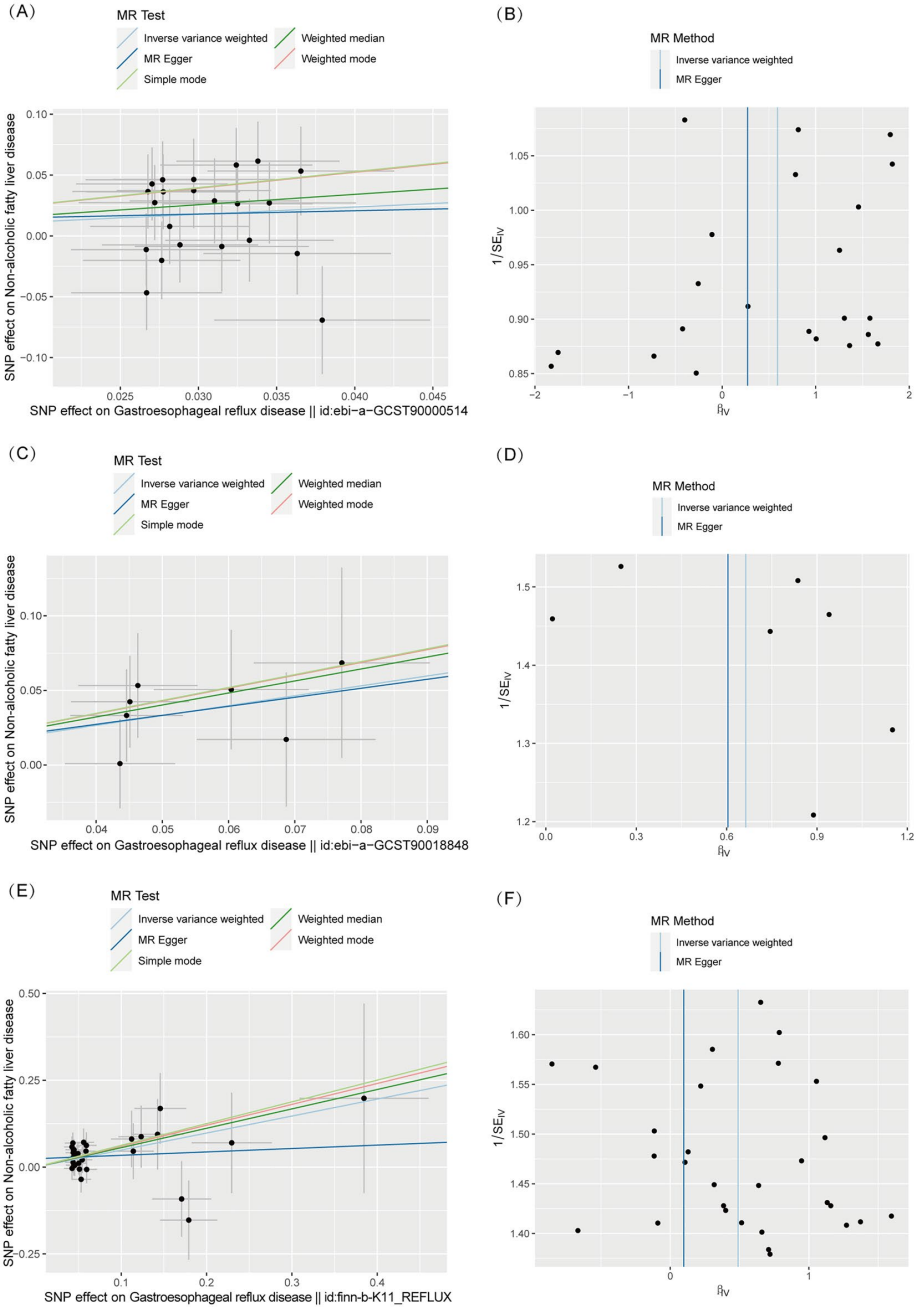
Scatter plots and Funnel plots of genetic associations with GERD against the genetic associations with NAFLD. (**A**) and (**B**) The scatter plot and funnel plot of the association between GERD (id: ebi-a-GCST90000514) and NAFLD. (**C**) and (**D**) The scatter plot and funnel plot of the association between GERD (id: ebi-a-GCST90018848) and NAFLD. (**E**) and (**F**) The scatter plot of the association between GERD (id: finn-b-K11_REFLUX) and NAFLD.

Leave-one-out cross-validation was utilized to calculate the MR result of the left IVs after removing them one by one. The β value remained above zero regardless of which genetic variants were removed, suggesting that each variant performed a positive influence on the outcome. With the assistance of the Steiger test, the direction of our analysis confirmed that there was no reverse causality (all *P* < 0.001) (Table 2, Figs. 2, 3 and Supplemental Fig. 1, 2, 3).

Of note, the issue of potential overlapping between databases (Group 2 and Group 3) is worth explaining. The exposure and outcome of Group 2^20^ may both have partial samples from FinnGen, while for Group 3 they both from FinnGen. The influence of potential overlapping (called weak instrument bias) can be measured by Type 1 error rates. We used the formulae of website built by Burgess et al.^32^ (https://sb452.shinyapps.io/overlap) to calculate the probability of making Type 1 error and the results showed that the rates of the two groups (Group 1, Group 2) were both approximately 5%, which could be acceptable and indirectly reflected a lower degree of overlap.

### Meta-analysis

**Figure 4:**
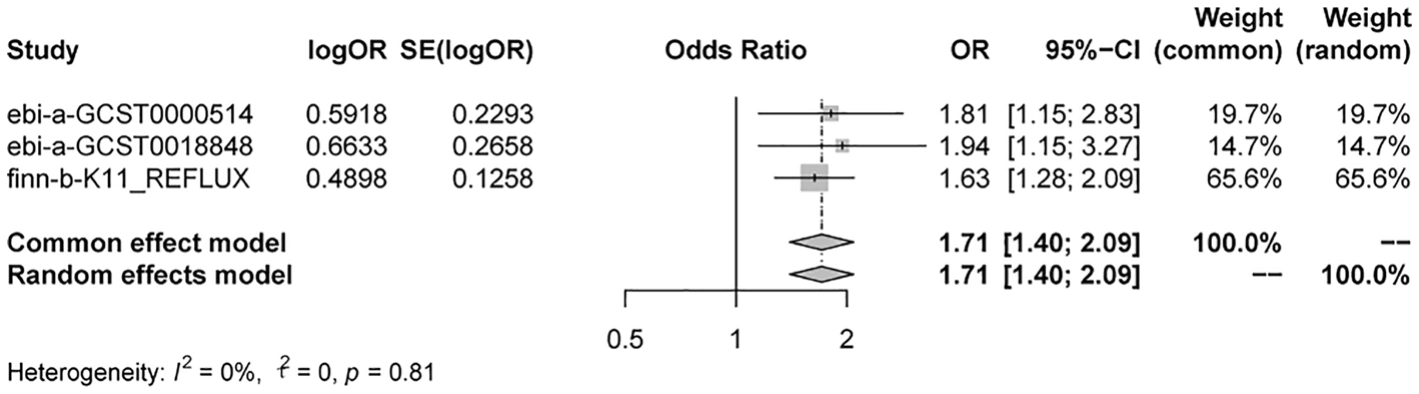
Forest plot of results from MR analysis.

Taking IVW as the major method, we conducted meta-analysis on it for an overall outcome. The result did not show any statistical heterogeneity (I^2^ = 0.0%; H = 1.00; *P* = 0.81). Accordingly, the fixed-effects model were chosen to perform the result (OR 1.71 95% CI 1.40–2.09; *P* < 0.0001) (Fig. 4 and Supplemental Table 7). Meanwhile, the Egger’s test (*P* = 0.26), Begg’s test (*P* = 0.33) and the visualization of funnel plot showed no evidence of publication bias (Supplemental Fig. 4).

## Discussion

To our knowledge, this study is the first MR analysis to reveal the causation between GERD and NAFLD. We found the overall risk of getting NAFLD is about 71% in people with GERD (OR 1.71 95% CI 1.40–2.09; *P* < 0.0001) in MR and meta analysis, by virtue of the GWAS and FinnGen databases^10^. This research is also consistent with Wijarnpreecha and Lee^13,33^.

The link between GERD and NAFLD has not been entirely understood. Several possible explanations may account for it.

There is evidence that people suffering from GERD are inclined to acquire insulin resistance (IR)^34^. The reflux of acid stimulates esophageal epithelial cells to produce chemokines, which subsequently activate various immune cells. Then these activated cells will produce myriad pro-inflammatory cytokines such as IL-6, IL-1, and TNF-alpha. On the one hand, these cytokines give rise to insulin resistance, making insulin lose its capability of inhibiting apoptosis. Utimately, NAFLD develops as a result of the accumulation of free fatty acids in the liver^35,36^. On the other hand, Cytokines tend to recruit Kupffer cells to intervene in inflammation and suppress lipid metabolism, ultimately leading to NAFLD^37^.

Reactive oxygen species (ROS) have been reported to play an important role in the pathogenesis of varied gastrointestinal (GI) diseases, including GERD and liver cirrhosis^38,39^. Acid exposure and sequential ROS generation will cause the damage to mucosal damage^35^.Simultaneously, ROS can be detrimental to the liver and accelerate the pathogenesis of NAFLD^40^.

There is evidence that visceral obesity, but not BMI, is closely correlated with the development of GERD and NAFLD^11,41–43^. The visceral adipose tissue (VAT) accumulated in the abdomen leads to the increase of intragastric pressure, which then contributes to abnormal acid reflux, and inescapably GERD^44,45^. Besides, the accumulation of free fatty acids in the liver and adipocytokine dysregulation induced by VAT will foster the chronic inflammation and promote the pathogenesis of NAFLD^11,46^.

Additionally, GERD patients tend to take PPIs, serving as the most effective treatment. In recent years, copious studies have reported that the long-term use of PPIs may induce the intestinal dysbiosis, affecting the composition of the gut microbiota and pushing the bacterial overgrowth^47^. On that occasion, the bacterial transit from luminal surface to the liver circulation is uplifted, thus causing inflammatory response in the liver and deteriorating its condition. At last, as mentioned above, inflammatory cytokines will produce NAFLD^48^.

## Limitation

However, some limitations still exist in the study. First, due to the lack of data on other races, the genetic variants of exposure and outcome were both retrieved from European ancestry. Thus, expanding the causal relationship discovered to other races was not appropriate, which suggested the lack of universality. However, the limited population frees the discovery from the bias of the different races of exposure and outcome. Second, although MR analysis avoids the drawbacks of observational studies, such as reverse causality and residual confounding, it is susceptible to pleiotropy. The use of the weighted median can solve this problem, because it works even if half of the SNPs are not valid^22,23^. Moreover, the MR-Egger intercept test is also joined to minimize the bias from pleiotropy. Third, despite that we removed the SNPs associated with confounders according to assumption (iii), this type of selection was based on the present GWAS database. We acknowledge that the analysis cannot control for unknown confounders, which may violate assumption (iii) and bias the analysis. Fourth, the study has only demonstrated that people with GERD are more likely to develop NAFLD, based on the existing databases. The specific mechanisms between the two diseases are warranting further examination. Finally, we used a dataset of currently searchable large samples for analysis. As the database increase and update, the result may change accordingly.

## Conclusion

In conclusion, our MR study combined with meta-analysis indicated the credible causal association between GERD and NAFLD. Considering the high prevalence of both diseases, further investigations may contribute to the development of new prevention and treatment strategies.

### Supplementary Information

Supplementary Legends.

Supplementary Tables.

Supplementary Figure 1.

Supplementary Figure 2.

Supplementary Figure 3.

Supplementary Figure 4.

## Data Availability

All the GWAS summary data in this study can be downloaded from the public repositories. The SNPs of GERD were from open EBI GWAS database (https://www.ebi.ac.uk/). The SNPs of otorhinolaryngological diseases could be retrieved from FinnGen database (http://www.finngen.fi/en).

## Abbreviations

CI: Confidence interval
GERD: Gastroesophageal reflux disease
GWAS: Genome-wide association study
GI: Gastrointestinal
IL: Interlukin
IR: Insulin resistance
IV: Instrumental variable
IVW: Inverse Variance Weighted
MR: Mendelian randomization
NAFLD: Non-alcoholic fatty liver disease
OR: Odds ratio
PPI: Proton pump inhibitor
ROS: Reactive oxygen species
SNP: Single nucleotide polymorphism
T2DM: Type II diabetes mellitus
TNF: Tumor necrosis factor

## References

[1] Gyawali CP, Kahrilas PJ, Savarino E (2018). Modern diagnosis of GERD: The Lyon Consensus. Gut.

[2] Azer SA, Reddivari AKR (2023). Reflux Esophagitis.

[3] Rettura F, Bronzini F, Campigotto M (2021). Refractory gastroesophageal reflux disease: A management update. Front. Med..

[4] Jung HK (2011). Epidemiology of gastroesophageal reflux disease in Asia: A systematic review. J. Neurogastroenterol. motil..

[5] El-Serag HB, Sweet S, Winchester CC, Dent J (2014). Update on the epidemiology of gastro-oesophageal reflux disease: A systematic review. Gut.

[6] Pouwels S, Sakran N, Graham Y (2022). Non-alcoholic fatty liver disease (NAFLD): A review of pathophysiology clinical management and effects of weight loss. BMC Endocr. Disord..

[7] Younossi Z, Anstee QM, Marietti M (2018). Global burden of NAFLD and NASH: Trends, predictions, risk factors and prevention. Nat. Rev. Gastroenterol. Hepatol..

[8] Huang TD, Behary J, Zekry A (2020). Non-alcoholic fatty liver disease: A review of epidemiology, risk factors, diagnosis and management. Intern. Med. J..

[9] Yang HJ, Chang Y, Park SK (2017). Nonalcoholic fatty liver disease is associated with increased risk of reflux esophagitis. Dig. Dis. Sci..

[10] Mikolasevic I, Poropat G, Filipec Kanizaj T (2021). Association between gastroesophageal reflux disease and elastographic parameters of liver steatosis and fibrosis: Controlled attenuation parameter and liver stiffness measurements. Can. J. Gastroenterol. Hepatol..

[11] Fujiwara M, Eguchi Y, Fukumori N (2015). The symptoms of gastroesophageal reflux disease correlate with high body mass index, the aspartate aminotransferase/alanine aminotransferase ratio and insulin resistance in Japanese patients with non-alcoholic fatty liver disease. Int. Med. (Tokyo, Jpn.).

[12] Miele L, Cammarota G, Vero V (2012). Non-alcoholic fatty liver disease is associated with high prevalence of gastro-oesophageal reflux symptoms Digestive and liver disease. Off. J. Ital. Soc. Gastroenterol. Ital. Assoc. Study Liver.

[13] Wijarnpreecha K, Panjawatanan P, Thongprayoon C, Jaruvongvanich V, Ungprasert P (2017). Association between gastroesophageal reflux disease and nonalcoholic fatty liver disease: A meta-analysis. Saudi J. Gastroenterol. Off. J. Saudi Gastroenterol. Assoc..

[14] Min YW, Kim Y, Gwak GY (2018). Non-alcoholic fatty liver disease and the development of reflux esophagitis: A cohort study. J. Gastroenterol. Hepatol..

[15] Catanzaro R, Calabrese F, Occhipinti S (2014). Nonalcoholic fatty liver disease increases risk for gastroesophageal reflux symptoms. Dig. Dis. Sci..

[16] Davey Smith G, Hemani G (2014). Mendelian randomization: Genetic anchors for causal inference in epidemiological studies. Hum. Mol. Genet..

[17] Bowden J, Holmes MV (2019). Meta-analysis and Mendelian randomization: A review. Res. Synth. Methods..

[18] Smith GD, Ebrahim S (2004). Mendelian randomization: Prospects, potentials, and limitations. Int. J. Epidemiol..

[19] Lee YH (2018). An overview of meta-analysis for clinicians. Korean J. Int. Med..

[20] Ong JS, An J, Han X (2022). Multitrait genetic association analysis identifies 50 new risk loci for gastro-oesophageal reflux, seven new loci for Barrett’s oesophagus and provides insights into clinical heterogeneity in reflux diagnosis. Gut.

[21] Sakaue S, Kanai M, Tanigawa Y (2021). A cross-population atlas of genetic associations for 220 human phenotypes. Nat. Genet..

[22] Dönertaş HM, Fabian DK, Valenzuela MF, Partridge L, Thornton JM (2021). Common genetic associations between age-related diseases. Nature aging..

[23] Sekula P, Del Greco MF, Pattaro C, Köttgen A (2016). Mendelian randomization as an approach to assess causality using observational data. J. Am. Soc. Nephrol. JASN.

[24] Burgess S, Thompson SG (2011). Avoiding bias from weak instruments in Mendelian randomization studies. Int. J. Epidemiol..

[25] VanderWeele TJ, TchetgenTchetgen EJ, Cornelis M, Kraft P (2014). Methodological challenges in mendelian randomization. Epidemiol. (Camb., Mass)..

[26] Didelez V, Sheehan N (2007). Mendelian randomization as an instrumental variable approach to causal inference. Stat. Methods Med. Res..

[27] Burgess S, Thompson SG (2017). Interpreting findings from Mendelian randomization using the MR-Egger method. Eur. J. Epidemiol..

[28] Burgess S, Foley CN, Allara E, Staley JR, Howson JMM (2020). A robust and efficient method for Mendelian randomization with hundreds of genetic variants. Nat. Commun..

[29] Hartwig FP, Davey Smith G, Bowden J (2017). Robust inference in summary data Mendelian randomization via the zero modal pleiotropy assumption. Int. J. Epidemiol..

[30] Xiao G, He Q, Liu L (2022). Causality of genetically determined metabolites on anxiety disorders: A two-sample Mendelian randomization study. J. Transl. Med..

[31] Chen X, Kong J, Diao X (2020). Depression and prostate cancer risk: A Mendelian randomization study. Cancer Med..

[32] Pierce BL, Burgess S (2013). Efficient design for Mendelian randomization studies: Subsample and 2-sample instrumental variable estimators. Am. J. Epidemiol..

[33] Lee S, Kim HM, Kim YJ, Moon CM, Cho JH, Han KJJG (2011). Erosive esophagitis is associated with fatty liver in school workers. Gastroenterology..

[34] Budiyani L, Purnamasari D, Simadibrata M, Abdullah M (2017). Differences in the insulin resistance levels measured by HOMA-IR between patients with erosive and non-erosive gastroesophageal reflux disease. J. ASEAN Fed. Endocr. Soc..

[35] Rehman K, Akash MSH, Liaqat A, Kamal S, Qadir MI, Rasul A (2017). Role of interleukin-6 in development of insulin resistance and type 2 diabetes mellitus. Crit. Rev. Eukaryot. Gene Expr..

[36] Cobbina E, Akhlaghi F (2017). Non-alcoholic fatty liver disease (NAFLD)—Pathogenesis, classification, and effect on drug metabolizing enzymes and transporters. Drug Metab. Rev..

[37] Watt MJ, Miotto PM, De Nardo W, Montgomery MK (2019). The liver as an endocrine organ-linking nafld and insulin resistance. Endocr. Rev..

[38] Kim YJ, Kim EH, Hahm KB (2012). Oxidative stress in inflammation-based gastrointestinal tract diseases: Challenges and opportunities. J. Gastroenterol. Hepatol..

[39] Ustaoglu A, Nguyen A, Spechler S, Sifrim D, Souza R, Woodland P (2020). Mucosal pathogenesis in gastro-esophageal reflux disease. Neurogastroenterol. Motil..

[40] Nassir F (2022). NAFLD: Mechanisms treatments and biomarkers. Biomolecules.

[41] Chang P, Friedenberg F (2014). Obesity and GERD. Gastroenterol. Clin. North Am..

[42] Chen X, Shi F, Xiao J (2022). Associations between abdominal obesity indices and nonalcoholic fatty liver disease: Chinese visceral adiposity index. Front. Endocrinol..

[43] Fujikawa Y, Tominaga K, Fujii H (2012). High prevalence of gastroesophageal reflux symptoms in patients with non-alcoholic fatty liver disease associated with serum levels of triglyceride and cholesterol but not simple visceral obesity. Digestion.

[44] Camilleri M, Malhi H, Acosta A (2017). Gastrointestinal complications of obesity. Gastroenterology.

[45] Nehlig A (2022). Effects of coffee on the gastro-intestinal tract: A narrative review and literature update. Nutrients.

[46] Milić S, Lulić D, Štimac D (2014). Non-alcoholic fatty liver disease and obesity: Biochemical, metabolic and clinical presentations. World J. Gastroenterol..

[47] Shi YC, Cai ST, Tian YP (2019). Effects of proton pump inhibitors on the gastrointestinal microbiota in gastroesophageal reflux disease. Genom. Proteom. Bioinform..

[48] Singh A, Garg R, Lan N, Siddiqui MT, Gupta M, Alkhouri N (2020). Association between anti-acid therapies and advanced fibrosis in type 2 diabetics with biopsy-proven non-alcoholic fatty liver disease. Indian J. Gastroenterol. Off. J. Indian Soc. Gastroenterol..

