# Supplementary material for: Gastroesophageal reflux disease and non-alcoholic fatty liver disease: a two-sample Mendelian randomization combined with meta-analysis
Source: Sci Rep. 2024 Jun 2;14:12633. doi: 10.1038/s41598-024-63646-z (PMC11144195; doi:10.1038/s41598-024-63646-z)
Supplement: Supplementary file 1 — Supplementary Legends. [file 41598_2024_63646_MOESM1_ESM.docx]

Supplementary Materials

***Supplementary tables legends:***

**Supplemental Table 1.** Description of the data sources of exposure (GERD)

**Supplemental Table 2.** Instrumental SNPs on gastroesophageal reflux disease and non-alcoholic fatty liver disease

**Supplemental Table 3.** Results of Mendelian randomization

**Supplemental Table 4.** Heterogeneity test of Mendelian randomization analysis

**Supplemental Table 5.** MR Egger intercept of Mendelian randomization

**Supplemental Table 6.** MR-PRESSO analysis

**Supplemental Table 7.** Results of Meta-analysis

***Supplementary figures legend:***

**Supplemental Figure 1.** MR leave−one−out sensitivity analysis for Gastroesophageal reflux disease (id:ebi-a-GCST90000514) on Non-alcoholic fatty liver disease

**Supplemental Figure 2.** MR leave−one−out sensitivity analysis for Gastroesophageal reflux disease (id:ebi-a-GCST90018848) on Non-alcoholic fatty liver disease

**Supplemental Figure 3.** MR leave−one−out sensitivity analysis for Gastroesophageal reflux disease (id:finn-b-K11_REFLUX) on Non-alcoholic fatty liver disease

**Supplemental Figure 4.** Visualization of funnel plot
