# Supplementary figures and images for: Gastroesophageal reflux disease and non-alcoholic fatty liver disease: a two-sample Mendelian randomization combined with meta-analysis
Source: Sci Rep. 2024 Jun 2;14:12633. doi: 10.1038/s41598-024-63646-z (PMC11144195; doi:10.1038/s41598-024-63646-z)

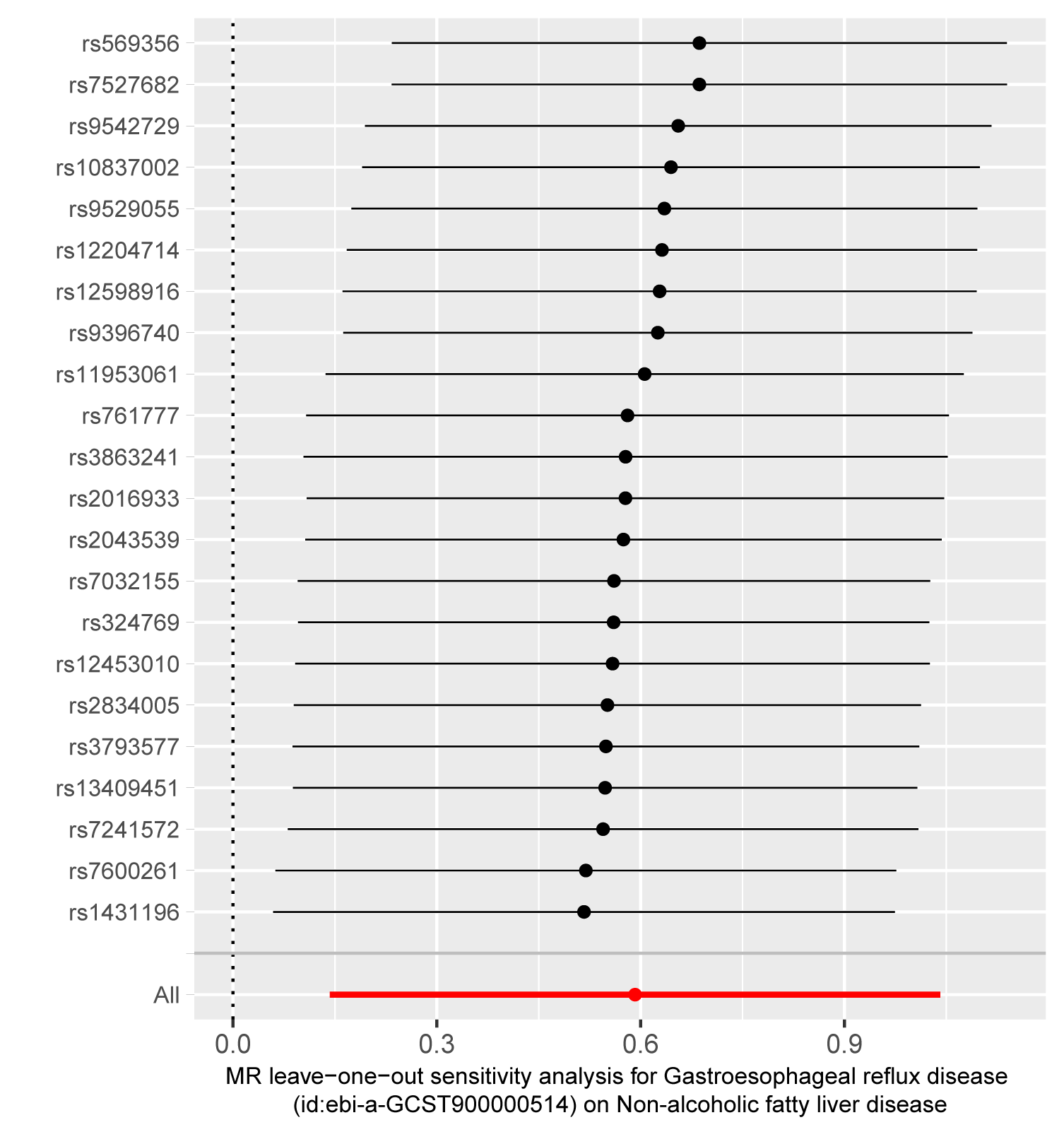

Supplement: Supplementary file 3 — Supplementary Figure 1. [file 41598_2024_63646_MOESM3_ESM.tif]

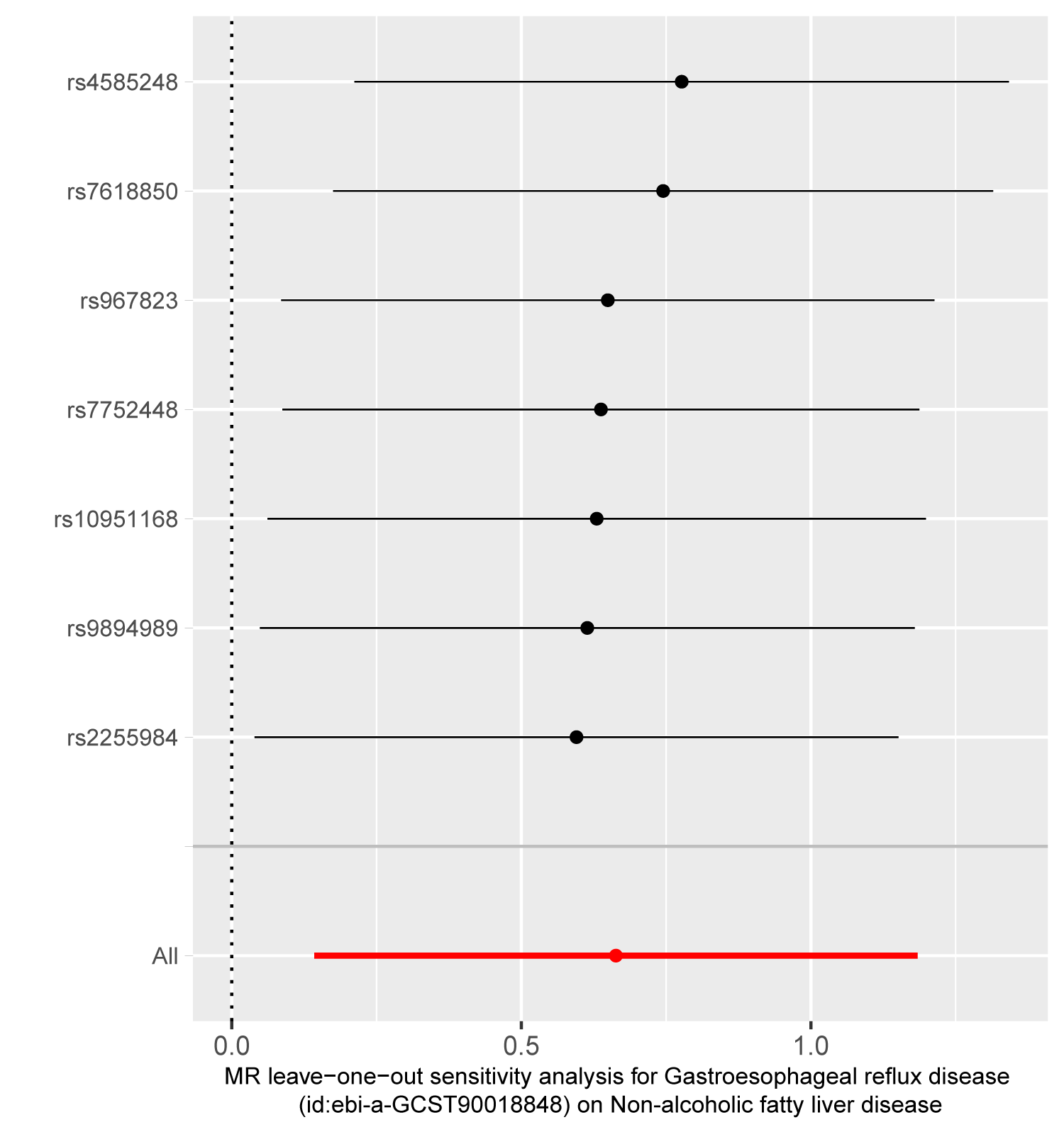

Supplement: Supplementary file 4 — Supplementary Figure 2. [file 41598_2024_63646_MOESM4_ESM.tif]

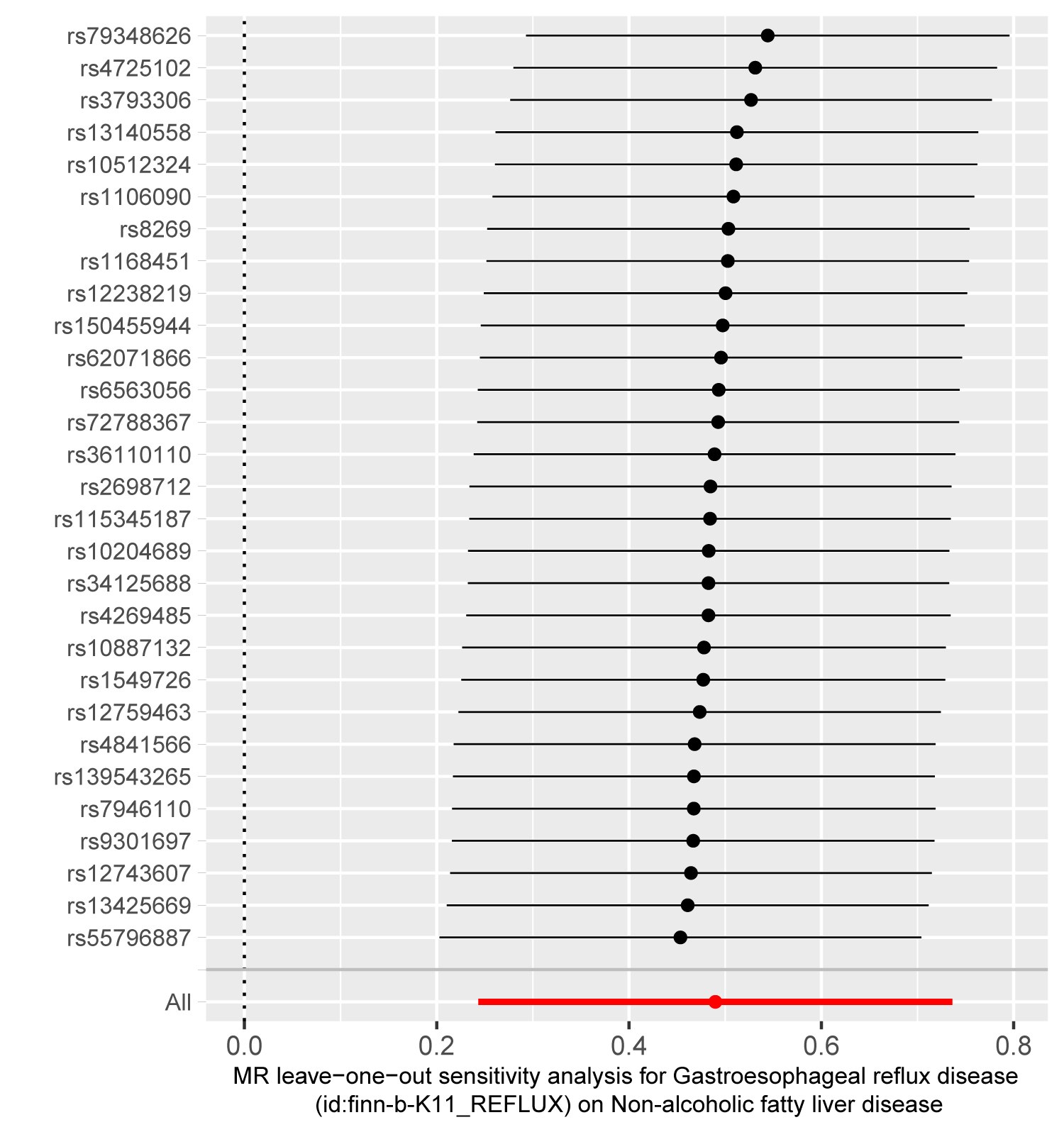

Supplement: Supplementary file 5 — Supplementary Figure 3. [file 41598_2024_63646_MOESM5_ESM.tif]

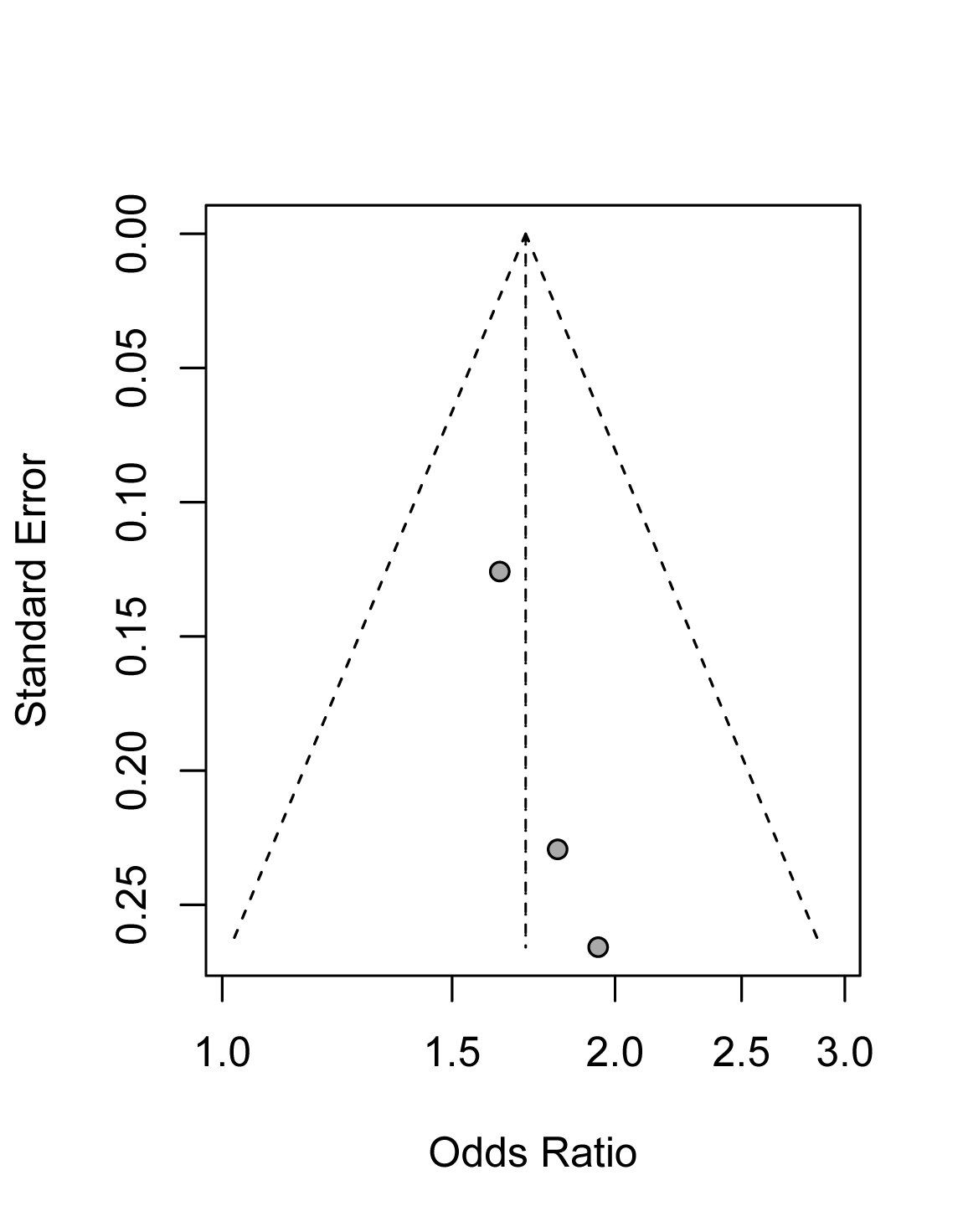

Supplement: Supplementary file 6 — Supplementary Figure 4. [file 41598_2024_63646_MOESM6_ESM.tif]
